# Multicentre phase II trial of trastuzumab and capecitabine in patients with HER2 overexpressing metastatic pancreatic cancer

**DOI:** 10.1038/bjc.2012.18

**Published:** 2012-02-28

**Authors:** J Harder, G Ihorst, V Heinemann, R Hofheinz, M Moehler, P Buechler, G Kloeppel, C Röcken, M Bitzer, S Boeck, E Endlicher, A Reinacher-Schick, C Schmoor, M Geissler

**Affiliations:** 1 Medizinische Klinik II, Hegau- Bodensee Klinikum, Virchowstraße 10, D-78224 Singen, Germany; 2 Clinical Trials Unit, University Medical Center Freiburg, Elsässer Straße 2, D-79110 Freiburg, Germany; 3 Klinikum Grosshadern Medizinische Klinik III, L.M.- Universitätsklinikum München, Marchoninistraße 15, D-81377 München, Germany; 4 Medizinische Klinik III, Klinikum Mannheim, Theodor-Kutzer-Ufer 1-3, D-68167 Mannheim, Germany; 5 Medizinische Klinik und Poliklinik, Klinikum der Johannes Gutenberg- Universität Mainz, Langenbeckstraße 1, D-55131 Mainz, Germany; 6 Abt. Allgemeine,- Viszerale-u. Unfallchirurgie, Chirurgische Universitätsklinik Heidelberg, Im Neuenheimer Feld 110, D-69120 Heidelberg, Germany; 7 Institut f. Pathologie, Christian-Albrechts-Universität, Arnold Heller-Straße 3, D-24105 Kiel, Germany; 8 Medizinische Klinik I, Universitätsklinikum Tübingen, Ottfried-Müller- Straße 10, D-72076 Tübingen, Germany; 9 Klinik und Poliklinik für Innere Medizin I, Klinikum der Universität Regensburg, Franz-Josef-Strauss-Allee 11, D-93042 Regensburg, Germany; 10 Hämatologie/Onkologie, Knappschaftskrankenhaus Bochum, In der Schornau 23–25, D-44892 Bochum, Germany; 11 Klinik für Allg. Innere Medizin, Onkologie/Hämatologie, Gastroenterologie u. Infektiologie, Klinikum Esslingen, Hirschlandstraße. 97, D-73730 Esslingen, Germany

**Keywords:** pancreatic cancer, immunohistochemistry, growth factors, chemotherapy

## Abstract

**Background:**

New therapeutic options for metastatic pancreatic cancer are urgently needed. In pancreatic cancer, overexpression of the epidermal growth factor receptor 2 (HER2) has been reported in up to 45%. This multicentre phase II study investigated the efficacy and toxicity of the HER2 antibody trastuzumab combined with capecitabine in the patients with pancreatic cancer and HER2 overexpression.

**Methods:**

Primary endpoint was progression-free survival (PFS) after 12 weeks. A total of 212 patients were screened for HER2 expression.

**Results:**

Immunohistochemical (IHC) HER2 expression was: 83 (40%) grade 0, 71 (34%) grade 1, 31 (15%) grade 2, 22 (11%) grade 3. A total of 17 patients with IHC +3 HER2 expression or gene amplification could be assessed for the treatment response. Grade 3/4 treatment toxicities were: each 7% leucopenia, diarrhoea, nausea and hand-foot syndrome. Progression-free survival after 12 weeks was 23.5%, median overall survival (OS) 6.9 months.

**Conclusion:**

This study demonstrates +3 HER2 expression or gene amplification in 11% of patients. Contrary to breast and gastric cancer, only 7 out of 11 (64%) patients with IHC +3 HER2 expression showed gene amplification. Although the therapy was well tolerated, PFS and OS did not perform favourably compared with standard chemotherapy. Together, we do not recommend further evaluation of anti-HER2 treatment in patients with metastatic pancreatic cancer.

Pancreatic cancer has a very poor prognosis, making it one of the five most common causes of cancer mortality in developed countries. After curative intended resection only 5–10% of patients with adenocarcinoma of the pancreas will be alive at 5 years after diagnosis. Advanced (unresectable locally advanced or metastatic) pancreatic cancer is an incurable disease with limited treatment options.

Since more than a decade, the nucleoside analogue gemcitabine is regarded as a standard of care for patients with advanced disease, providing clinical benefit and a moderate improvement in survival (Burris *et al*, 1997). Several randomised phase III trials failed to show a survival benefit for gemcitabine-based combination chemotherapy so far; however, meta-analytic data suggest a possible survival benefit for the use of fluoropyrimidines including capecitabine in combination with gemcitabine in selected patients, that is, those with metastatic disease and a good performance status (Heinemann *et al*, 2008). The anti-EGFR tyrosine kinase inhibitor erlotinib was demonstrated to result in superior survival, especially in patients developing skin rash (Moore *et al*, 2007). HER2 is a related receptor tyrosine kinase encoded by proto-oncogenes. Once activated, the signal-transduction cascade promotes cellular proliferation migration and survival. Immunohistochemical (IHC) overexpression of HER2 in various tumour cells has been not only associated with a poor prognosis, but also offers the therapeutic option of receptor targeting therapies. Studies report HER2 expression in up to 45% of patients with pancreatic cancer (Yamanaka *et al*, 1993). Targeting HER2 in pancreatic cancer cell lines and a xenograft mouse model showed encouraging results (Buechler *et al*, 2001, 2005).

Therefore, the aim of this study was to clarify the clinical significance of HER2 expression in patients with metastatic pancreatic cancer and to determine the potential of HER2 as therapeutic target in these patients.

On the basis of these data, we assessed the activity of the combination of trastuzumab and capecitabine in patients with advanced IHC +3 HER2 expressing pancreatic cancer or HER2 gene amplification. As the outcome of patients with locally advanced and metastatic pancreatic cancer is different and the response assessment in the latter is more reliable, only patients with metastatic disease were included. The objectives of the trial were to determine progression-free survival (PFS) after 12 weeks (primary endpoint), PFS, overall survival (OS), response rate (according to RECIST criteria), clinical benefit response (CBR) and safety profile.

## Patients and methods

### Patients and treatment

This was a prospective, single-arm, open-label, multi-centre phase II trial to investigate the efficacy and safety of trastuzumab (Herceptin) and capecitabine (Xeloda) as first-line treatment in patients with IHC +3 HER2 expressing advanced pancreatic cancer or cancer with HER2 gene amplification of stage IVB (T_1−4_N_0−1_M_1_).

Patients’ inclusion and exclusion criteria are shown in Table 1. Patients fulfilling the main inclusion and exclusion criteria were screened for HER2 expression. Patients were included into the trial in case of IHC +2 HER2 expression (additionally confirmed by gene amplification, fluorescence *in situ* hybridisation (FISH)) or +3 HER2 expression.

Patients received 4 mg kg^−1^ trastuzumab at first infusion followed by weekly 2 mg kg^−1^ combined with 1250 mg m^−2^ capecitabine twice daily on days 1–14 of a 3-week cycle. Treatment was continued until disease progression.

The study was approved by the institutional review board and ethics committee of each participating centre, and informed consent was given by each patient according to the Declaration of Helsinki. The trial is registered with WHO primary register number DRKS00000600.

### Procedures

#### Immunohistochemistry

Immunohistochemistry was performed on adjacent deparaffinised freshly cut sections using the peroxidase-labelled streptavidin–biotin technique, Dako REAL detection system (Glostrup, Denmark) for HER2. All immunostaining was performed in strict accordance with the FDA-approved REAL detection system package (Dako). Immunohistochemical results were scored independently by two pathologists ‘blind’ to all case data. Additional tissue controls were performed along with the included cell line controls.

Detection of HER2 was performed with heat-induced epitope retrieval and the use of the anti-HER2 primary antibody. Immunohistochemical staining was performed using the Dako Autostainer. The percentage of carcinoma cells was estimated in categories negative, weak (1+), moderate (2+) or intense (3+) if 10% or more of carcinoma cells attain plasma membrane staining. Cytoplasmic staining was discounted.

#### Fluorescence *in situ* hybridisation

The PathVysion detection kit (Abbott Laboratories, Abbott Park, IL, USA) was used for FISH analysis in case of IHC +2 HER2 expression. Previous studies showed in breast and colorectal cancer but also in biliary malignancies, which are biologically related to pancreatic cancer, that +3 HER2 expression is regular induced via gene amplification (Kobayashi *et al*, 2002; Ooi *et al*, 2004; Carlson *et al*, 2006; Yau *et al*, 2008; Harder *et al*, 2009). Therefore, we did not perform FISH analysis in +3 HER2 expressing tumour specimens before treatment with trastuzumab.

FISH-stained sections were scanned at × 1000 magnification and three separate carcinoma areas identified. Twenty nuclei were assessed in each area; HER2 and chromosome 17 copy numbers were counted for all cells and the ratio of *HER2* to chromosome 17 was calculated. Normal mean *HER2* : 17 ratio was defined as <2; a ratio >2 was taken as gene amplification. Normal mean *HER2* copy number was taken as <4 signals per cell.

In order to explain the treatment results we retrospectively analysed 11 out of 17 tumour specimens for HER2 gene amplification from patients with IHC +3 HER2 expression who were treated by trastuzumab and capecitabine. Six could not be analysed due to various reasons (mainly cytological samples or to small sample size).

The retrospective testing used the *HER2*-SISH double labelling *in situ* hybridisation system, and the Ventana BenchMark XT automated slide staining system (all Roche Diagnostics GmbH, Mannheim, Germany). Gene amplification was assessed according to the breast cancer scoring system (Rüschoff *et al*, 2009).

### Endpoints

Tumour response was evaluated by CT or MRI every 2 cycles (every 6 weeks) and classified as complete remission (CR), partial remission (PR), stable disease, progressive disease (PD) according to RECIST (Therasse *et al*, 2000).

Primary endpoint was the PFS rate 12 weeks after the start of treatment. Secondary endpoints were PFS time, OS time, time to response (CR/PR), duration of response, CBR 12 weeks after the start of treatment and quality of life (QOL) using the EORTC QLQ-C30 QOL Questionnaire. Clinical benefit response is a composite endpoint assessing the improvement in pain (pain intensity and analgesic consumption) and Karnofsky performance status as primary measures and integrating body weight as a secondary measure. Patients were classified as responders, if pain or Karnofsky performance status was improved or, in case of stability of pain and Karnofsky performance status, body weight was increased (Burris *et al*, 1997).

PFS was defined as the time from beginning of chemotherapy to disease progression or death, whichever occurred first. Overall survival time was defined as the time from beginning of chemotherapy to death.

Toxicity was evaluated according to NCI/CTC version 3.0 of 12 December 2003.

### Statistical analysis

Sample size calculation was based on the primary endpoint PFS rate 12 weeks after the start of treatment. The number of patients to be included was determined by the two-stage design according to Simon (1989) and was based on the following considerations. After monotherapy with capecitabine, median PFS time is 2.8 months (Cartwright *et al*, 2002). On the basis of these results, treatment with trastuzumab and capecitabine is considered to be not sufficiently active if the PFS rate 12 weeks after the start of treatment is 50% or lower. Treatment with trastuzumab and capecitabine is considered to be promising for further evaluation if the PFS rate 12 weeks after start of treatment is 70% or higher.

In a first step, 23 patients should be included and treated in the study. If 12 or less of these 23 patients were alive and free of PD after 12 weeks, the study should be stopped and the treatment considered ineffective in this group of patients. If at least 13 patients were alive and free of progression after 12 weeks of treatment, recruitment should be continued until 37 patients were included. If 24 or more of these 37 patients were alive and free of progression after 12 weeks, treatment should be considered as promising and should be studied further. This procedure ensures that if the treatment has a true PFS rate of at least 70%, the chance of erroneously rejecting the regime is ⩽20%. The chance of erroneously considering the treatment effective is ⩽5% if the true PFS rate is ⩽50%.

The primary efficacy analysis was based on the full analysis set (FAS), including all patients for whom treatment was started. An additional analysis was performed in the per protocol (PP) population with those patients who had received at least two complete cycles of chemotherapy, or had terminated treatment due to toxicity, early progression or death before day 43. The PFS rate and the OS rate were estimated by the Kaplan–Meier method. The effect of CA 19-9 serum concentration was investigated with a Cox regression model. Here, a cutpoint of 1000 U ml^−1^ for CA 19-9 was chosen as value close to the median 796 U ml^−1^ in order to obtain two patient groups of approximately the same size.

The safety analysis set (SAF) was defined as those patients who received at least one dose of chemotherapy. The SAF is identical to the FAS in this study, therefore no further distinction will be made. The incidence of toxicity and adverse events (AEs) was calculated as the number of patients who experienced at least one toxicity/AE of a certain category as a percentage of the total number of patients.

SAS software (SAS Institute Inc., Cary, NC, USA) version 9.2 was used for the analysis.

## Results

Between July 2004 and May 2008, a total of 212 patients were screened for HER2 expression and eligibility criteria at nine institutions. In 207 patients, the tumour specimens could be assessed for HER2 expression and gene amplification. In IHC 83 (40%) were grade 0, 71 (34%) grade 1, 31 (15%) grade 2 and 22 (11%) grade 3, respectively. One tumour specimen with IHC grade 2 showed gene amplification. In the initial assumption of the study protocol all IHC grade 3-positive specimens were considered FISH positive (*HER2* to chromosome 17 ratio >2) and treated with trastuzumab if there were no other exclusion criteria. In a *post hoc* analysis, taking into account the low response rates, we analysed 11 out of 17 IHC +3 HER2 expressing samples from patients who were treated with trastuzumab and capecitabine for HER2 gene amplification. Seven tested positive (64%) and four were negative.

Of 23 patients with IHC +3 HER2 expression or IHC +2 HER2 expression and HER2 gene amplification, 11 (47.8%) had a CA 19-9 value at screening ⩾1000 U ml^−1^, whereas among 163 patients without HER2 gene amplification, 74 (45.4%) CA 19-9 values were <1000 U ml^−1^.

The study was closed prematurely due to low HER2 expression and slow recruitment after screening 212 patients, 97 women (45.8%); median age 64 years, range 38–86 (Table 2).

**Figure 1:**
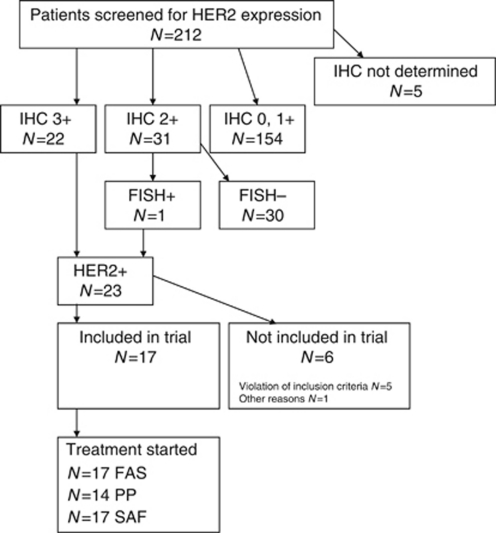
Flow diagram of patient recruitment. Abbreviations: FAS=full analysis set; PP=per protocol; SAF=safety analysis set.

From the 23 patients with IHC +3 HER2 expression or IHC +2 and HER2 gene amplification, 17 patients from four centres were included in the study and could be assessed for response to treatment in the full analysis set, 8 women (47.1%); median age 64 years, range 42–77 (Table 1). Five patients were not included because of violated inclusion/exclusion criteria. One patient was not included in the trial for other reasons (Figure 1).

Patients received a median number of three cycles (range 1–23), a median weekly trastuzumab dose of 145 mg (range 91–330), and a median weekly capecitabine dose of 15,429 mg (range 4500–31250), relating to median 99.4% of the planned trastuzumab dose (range 64.4–118.2) and median 77.9% of the planned capecitabine dose (range 19.3–148.8).

**Figure 2:**
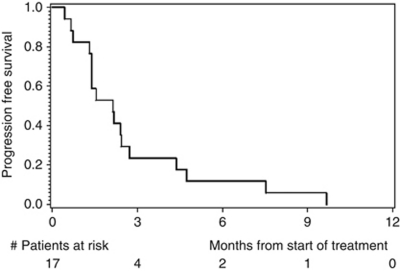
Kaplan–Meier estimates of progression-free survival.

**Figure 3:**
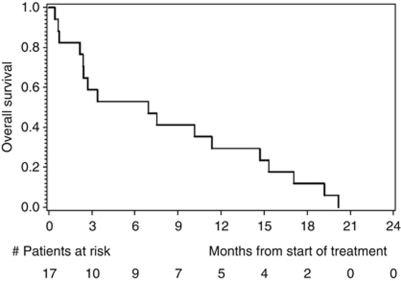
Kaplan–Meier estimates of overall survival.

Disease progression was observed in 13 out of 17 patients after 12 weeks of treatment, and the primary endpoint PFS rate after 12 weeks was thus estimated as 23.5% (exact 95% CI: 6.8–49.9). With only four patients alive and free of PD after 12 weeks, the trial would have been stopped for futility in an interim analysis planned after the inclusion of 23 patients. Median PFS was 65 days, with an estimated PFS rate after 6 months of 11.8% (95% CI: 0–27.1) and after 12 months of 0% (Figure 2). Median OS was 6.9 months, with an estimated OS probability after 6 months of 52.9% (95% CI: 29.2–76.7), and after 12 months of 29.4% (95% CI: 7.8–51.1; Figure 3). There was no statistical correlation between treatment response (PFS after 12 weeks) and HER2 gene amplification.

Analysis of CBR was planned in the study protocol for those patients with pain intensity ⩾2 on a visual analogue scale or use of ⩾70 mg of morphine equivalents the week prior inclusion and Karnofsky performance index <80 before start of treatment (Burris *et al*, 1997). One patient fulfilled these criteria, which died after 13 days, showing no CBR.

The impact of CA 19-9 serum concentration on PFS was investigated with a Cox proportional hazards regression model. The hazard ratio was estimated as 1.37 (95% CI: 0.50–3.72; *P*=0.54) for CA 19-9 ⩾1000 U ml^−1^ at screening compared with lower CA 19-9 levels.

The analysis of the primary endpoint was repeated in the PP population, where 10 out of 14 patients had disease progression after 12 weeks of treatment (estimated PFS rate: 28.6%, exact 95% CI: 8.4–58.1).

Two patients died early after 13 and 20 days, respectively, for these patients no toxicity data were documented.

Reported grade 3/4 toxicities in 15 patients with 88 cycles of chemotherapy were: leucopenia 1 out of 15 (6.7%), anaemia 0%, thrombocytopenia 0%, diarrhoea 1 out of 15 (6.7%), nausea 1 out of 15 (6.7%), hand–foot syndrome 1 out of 15 (6.7%). There had been no trastuzumab-attributable cardiac toxicity.

A total number of 101 AEs were observed, relating to an average number of 5.9 AEs per patient, range 0–17. Of these, 16 AEs were considered as serious (SAE). Most frequent AEs were of gastrointestinal origin (12 out of 17, 70.6%), general health problems as fatigue, (9 out of 17, 52.9%) and infections in 6 out of 17 patients (35.3%).

## Discussion

The expression of the growth factor receptors HER2 has been studied in different tumour types leading to the standard therapeutic use in breast cancer. Recently trastuzumab in combination with chemotherapy was considered as a new standard option for patients with HER2-positive advanced gastric or gastro–oesophageal junction cancer (Bang *et al*, 2010).

In other cancers drugs such as trastuzumab and lapatinib are under investigation. There are data of HER2 expression in up to 45% of patients with pancreatic cancer in mainly small cohorts (Yamanaka *et al*, 1993). Therefore, this study was designed to investigate the combination of trastuzumab and capecitabine as palliative first-line therapy in patients with metastatic pancreatic cancer. Beside the report by Yamanaka *et al*, there are two reports by Safran *et al* (2001, 2004) who first screened 154 patients with pancreatic cancer and showed HER2 overexpression in 21% (Safran *et al*, 2001) and 16% (Safran *et al*, 2004) of patients, respectively. Thirty-four patients were treated with trastuzumab and gemcitabine because the tumours showed HER2 overexpression (+2 HER2 in 30 and +3 HER2 in 4 tumours (Safran *et al*, 2004)). From the 21% of 154 patients (Safran *et al*, 2001), 23 (15%) had IHC +2 HER2 expressing tumours and 9 (6%) +3 HER2 expression, examined by standardised methods. In 3 out of 8 (40%) IHC +2 HER2 and 0 out of 3 +3 HER2 expressing tumours they could show gene amplification by FISH. In the second cohort, they report HER2 amplification in 2 out of 13 and hyperploid chromosome 17 in 3 out of 13 specimens with IHC HER2 overexpression.

This multi-centre study demonstrates IHC +2 HER2 expression in 31 out of 207 (15%) and +3 HER2 expression in 22 out of 207 (11%) of patients with metastatic pancreatic cancer. Only 7 out of 11 (64%) patients with IHC +3 HER2 expression showed gene amplification and 1 out of 22 (5%) with +2 HER expression.

The data for IHC HER2 expression reported by Safran *et al* and from this study are comparable except for HER2 gene amplification in IHC +2 HER2 expressing tumours. One explanation therefore might be the different test method used for the first cohort reported by Safran *et al* (2001). In the second cohort they summarised gene amplification for IHC +2 and +3 HER2 expression and counted hyperdiploid chromosome 17 as FISH positive, irrespective of the ratio HER2 to chromosome 17. Second they (Safran *et al*, 2004) used CT-guided biopsies or fine-needle aspirates, whereas in our study most samples were true cut biopsies. The sample quality might be important especially for FISH. These two points might explain the difference in FISH positivity between current series and Safran *et al* especially in IHC +2 HER2 expressing tumours. In summary, reports of HER2 expression, not only in pancreatic cancer, are confusing as the term ‘overexpression’ was also used for +1 HER2 immunostaining (Yamanaka *et al*, 1993). As the example from HER2 IHC shows there is a strong need for consistent and accurate reporting of biomarker data.

Breast, colorectal and biliary cancer shows high concordance between HER2 expression and amplification (Ooi *et al*, 2004; Yau *et al*, 2008; Harder *et al*, 2009). Furthermore, HER2 amplification is associated with treatment response (Hudis, 2007). In gastric cancer a new histological scoring system for HER2 IHC was established in the ToGA trail (Bang *et al*, 2010). Additional HER2 gene amplification was found in 13% and 27% in IHC +1 and +2 tumours, respectively, although almost all gastric tumours with IHC +3 HER2 expression showed gene amplification.

In a *post hoc* analysis, we performed FISH for gene amplification in +3 HER2 expressing tumours in order to explain the conflicting data regarding HER2 amplification in IHC +2 HER2 tumours and the poor treatment results found in this study. In accordance with Safran *et al* this study shows no consistent HER2 gene amplification in IHC +3 expressing pancreatic adenocarcinoma as seen in other cancers, suggesting some other pathways resulting in protein overexpression. Contrary to breast, colon, biliary and gastric cancer in pancreatic cancer IHC +2 HER2 expression and especially +3 HER2 expression does not correlate with gene amplification. May be HER2 overexpression in pancreatic cancer is due to gene deregulation rather than gene amplification as postulated by Ukita *et al* (2002) for intrahepatic biliary tract cancer.

Due to the low incidence of IHC +3 HER2 expression, only 17 patients could be treated with trastuzumab and capecitabine in this trial. Although the therapy was well tolerated, and PFS and OS are comparable to previous regimens, the combination of trastuzumab and capecitabine did not perform favourably compared with historical standard gemcitabine or capecitabine chemotherapy.

The RR reported by Safran *et al* (2004) was 24% overall and even 34% for patients with HER2 gene amplification resulting in a median OS of 7.5 months. These encouraging results led to our study design and numbers calculated. Gemcitabine is considered as the chemotherapy of choice for metastatic pancreatic cancer. There is no comparative trial between Gemcitabine and Capecitabine, but Capecitabine has to be considered to have superior response rates over bolus 5-FU and similar to Gemcitabine (Burris *et al*, 1997; Cartwright *et al*, 2002). Therefore, we designed this study in intention for a patient-friendly protocol including an oral chemotherapy. In addition there was the hope that combination therapy including capecitabine might result in better response rates taking into account the disappointing results using gemcitabine in combination with anti-growth factor therapies (Xiong *et al*, 2004). Median PFS in our trial was 65 days with an OS of 6.9 month, which is similar to single-agent capecitabine (Cartwright *et al*, 2002). In a recent study, published in abstract form (McDermott *et al*, 2011) capecitabine in combination with lapatinib, a small molecule, tyrosine kinase inhibitor of epidermal growth factor receptor and HER2 showed disappointing antitumour activity with an OS of 4 months. Our study showed an median OS of 6.9 months, but taken together capecitabine in combination with anti-HER2 therapy does not seem to significantly improve treatment results in comparison with historical capecitabine monotherapy (mean OS 6.0 months; Cartwright *et al*, 2002). Therefore, a comparative trial can not be recommended.

That one-third of IHC 3+ HER2-positive tumours showed no HER2 amplification found in a *post hoc* analysis might be one explanation for the disappointing treatment results adding anti-HER2 treatment to capecitabine. The phenomenon that targeted therapy containing regimens are ineffective in pancreatic cancer while the same regimens have shown significant activity in other GI tumour entities is also known for anti-EGFR and anti-VEGF drugs (Kindler *et al*, 2010; Philip *et al*, 2010). Therefore, it is also unlikely that the combination of trastuzumab and cetuximab will show synergistic effects in human pancreatic cancer as suggested by a xenograft mouse model (Larbouret *et al*, 2010). There may be downstream signalling events involved in non-responsiveness to trastuzumab such as RAF, PI3K including the presence of k-ras mutations, which can be found in 60–70% of pancreatic cancer patients. In addition, IGF and or TGFb signalling may overcome the effect of HER2 targeting (Vaccaro *et al*, 2011). Additional, tumour-associated fibroblasts and stromal development may counteract the effect of chemotherapy including targeted therapy. The heterogeneous and complex biology of pancreatic cancer or some specific tumour–stroma interaction, in a cancer with a strong desmoplastic environment, might are some reasons why many chemotherapy regimens containing an anti-growth factor inhibitor failed to significantly improve OS. According to recent reports one promising way to overcome chemotherapy resistance in pancreatic cancer is to focus on the tumour environment and tumour stem cells (Bednar and Simeone, 2011; Liles *et al*, 2011).

There are limitations of this study. First, we miscalculated the needed study sites for rapid patient recruitment as shown by Bang *et al* for gastric cancer. Therefore, the study had to be closed prematurely. Second, erroneous we did not perform FISH for IHC +3 HER2 expressing tumours prospectively. Relying on the data found for breast, colon and biliary cancer we assumed HER2 amplification in +3 HER2 expressing tumours. Third, there was no control group comparing anti-HER2 treatment with conventional chemotherapy because the study was designed as a two-step phase II study. This way testing chemotherapy regiments with only a few study sites does not fit for biomarker-guided trials studying targeted therapy applicable to 10–20% of patients.

Despite these limitations, we can conclude that IHC +2 and +3 HER2 expression is present in about 25% but HER2 gene amplification in only 4% of patients with pancreatic cancer. Even in patients with HER2 amplifying tumours, the combination chemotherapy with trastuzumab and capecitabine does not result in improved PFS and OS compared with historical gemcitabine or capecitabine alone. According to these results we do not recommend further evaluation of anti-HER2 treatment in patients with metastatic pancreatic cancer.

## Figures and Tables

**Table 1:** Inclusion and exclusion criteria

**Inclusion criteria**	**Exclusion criteria**
Age⩾18 years	Eligibility for surgery or neoadjuvant radiotherapy with curative intent
At least one measurable lesion ⩾2 cm with CT or MRI	Pre-existing peripheral neuropathy
No previous palliative chemotherapy or radiotherapy	Symptomatic cerebral metastases
Performance status 0–2 according to WHO/ECOG or Karnofsky status ⩾60	Incompatibility with or allergy to the antineoplastic agents used
Life expectancy of at least 3 months	Known DPD insufficiency
Adequate organ function (absolute neutrophiles ⩾1.5 × 10^9^ per l, haemoglobin ⩾80 g l^−1^, platelet count ⩾100 × 10^9^ l^−1^, total bilirubin ⩽3 × upper limit of normal, creatinine clearance ⩾30 ml min^−1^, AST and ALT ⩽2.5 × upper limit of normal, <5 × upper limit of normal in case of liver metastases)	Additional malignancies other than completely excised *in situ* carcinoma of the cervix or non melanomatous skin cancer
LVEF >50%	Pregnancy or lactation
Informed consent	Serious infection
	Current alcohol or drug addiction
	Known DPD insufficiency

Abbreviations: ALT=alanine aminotransferase; AST=aspartate aminotransferase; CT=computed tomography; DPD=dihydropyrimidindehydrogenase; ECOG=Eastern Cooperative Oncology Group; LVEF=left ventricular ejection fraction; MRI=magnetic resonance imaging; WHO=World Health Organization.

**Table 2:** Baseline patient characteristics

	**Patients screened (*N*=212)**	**Patients included (*N*=17)**
Age at registration (years): median (range)	64 (38–86)	64 (42–77)
		
*Sex:* n (%)		
Female	97 (45.8)	8 (47.1)
		
*Tumour stage at first diagnosis:* n (%)		
I	3 (1.6)	0 (0)
II	16 (8.7)	2 (11.8)
III	60 (32.6)	4 (23.5)
IV	47 (25.5)	7 (41.2)
Not evaluable	58 (31.5)	4 (23.5)
Not available	28	—
		
*Adenocarcinoma:* n (%)		
Yes	202 (98.1)	17 (100)
No	4 (1.9)	0
Not available	6	—
		
*IHC/FISH:* n (%)		
0	83 (40.1)	0
1+	71 (34.3)	0
2+/FISH−	30 (14.5)	0
2+/FISH+	1 (0.5)	1 (5.9)
3+	22 (10.6)	16 (94.1)
Not available	5	—
		
CA 19–9 (U ml^−1^), median (range); not available: *n*=22	766 (0–190, 953)	796 (8–130, 603)

Abbreviations: CA=carbohydrate antigen; FISH=fluorescence *in* *situ* hybridisation; IHC=immunohistochemistry.
